# Deep GONet: self-explainable deep neural network based on Gene Ontology for phenotype prediction from gene expression data

**DOI:** 10.1186/s12859-021-04370-7

**Published:** 2021-09-22

**Authors:** Victoria Bourgeais, Farida Zehraoui, Mohamed Ben Hamdoune, Blaise Hanczar

**Affiliations:** IBISC, Univ Evry, Université Paris-Saclay, 91020 Évry-Courcouronnes, France

**Keywords:** Gene expression, Phenotype prediction, Model interpretation, Deep learning, Gene Ontology

## Abstract

**Background:**

With the rapid advancement of genomic sequencing techniques, massive production of gene expression data is becoming possible, which prompts the development of precision medicine. Deep learning is a promising approach for phenotype prediction (clinical diagnosis, prognosis, and drug response) based on gene expression profile. Existing deep learning models are usually considered as black-boxes that provide accurate predictions but are not interpretable. However, accuracy and interpretation are both essential for precision medicine. In addition, most models do not integrate the knowledge of the domain. Hence, making deep learning models interpretable for medical applications using prior biological knowledge is the main focus of this paper.

**Results:**

In this paper, we propose a new self-explainable deep learning model, called Deep GONet, integrating the Gene Ontology into the hierarchical architecture of the neural network. This model is based on a fully-connected architecture constrained by the Gene Ontology annotations, such that each neuron represents a biological function. The experiments on cancer diagnosis datasets demonstrate that Deep GONet is both easily interpretable and highly performant to discriminate cancer and non-cancer samples.

**Conclusions:**

Our model provides an explanation to its predictions by identifying the most important neurons and associating them with biological functions, making the model understandable for biologists and physicians.

## Background

With the rapid advances of data acquisition technologies, collecting large amounts of different-type data (images, ECG, genomics...) becomes simpler in the medical field. It inspires a new form of this field, i.e., precision medicine, which takes advantage of these available data to improve profoundly diagnosis, prognosis, or therapeutic decision. Precision medicine has access to detect in advance a disease, such as cancer, anticipate the progression of the disease, and adapt the therapy according to the characteristics of patients. Among these data, genomic data and especially gene expression data play a key role in the development of precision medicine. Gene expression profile is known to be an indicator of the cellular state and allows the study of complex diseases.

For many years, machine learning has been used on transcriptomic data to construct classifiers predicting phenotypes (diagnosis, prognosis, treatment) [1]. In the last decade, deep learning has become the source of the most impressive improvements in machine learning [2]. It shows its superiority in many domains such as image analysis or natural language processing. Its main advantage is that it constructs high levels of data abstraction by stacking multiple linear and non-linear processing units. Deep learning has recently been applied to classification based on gene expression problems. Unlike images or texts, gene expression data have no structure. The architectures used in the literature are, therefore, mainly autoencoders and multilayer perceptrons (MLP) [3]. For instance, *Stacked Denoising Autoencoders* [4, 5] are exploited to extract a lower dimension from the data, then a classifier (such as support vector machine (SVM) or MLP) is applied to perform classification. MLP are used in [6, 7] to predict directly diseases without dimension reduction. Despite promising first results, deep learning has not made a breakthrough in gene expression classification yet because of the often small size of the available training sets. Deep learning is especially good with large training sets. In the next years, with the increasing production of transcriptomic data, it is highly likely that deep learning will play a major role to solve these problems.

One of the main concerns of the application of deep learning in the medical field is its lack of interpretability. Indeed, the neural networks are black-box models, which means that the model cannot provide an explanation to its decision. The interpretation of machine learning algorithms is one of the most essential topics nowadays, especially in the case of medical application for three main reasons. First, both the physician and his patient must understand why the model predicts a given phenotype. Particularly, it can influence later decisions such as the choice of the treatment. Second, it is important to ensure that the model bases its predictions on a reliable representation of the data and does not focus on irrelevant artifacts. This will highly impact the trust of the physicians toward the predictions regardless of the performances of the model. Finally, the model with high-accurate predictions may have identified interesting patterns that biologists would like to investigate.

We can distinguish two main approaches for interpreting the black-boxes: the post-hoc methods and the self-explainable models [8]. In a post-hoc method, the black-box model is first learned and then an interpretation method is used to explain the predictions. Several post-hoc methods with different purposes are proposed in the literature. Among them, proxy methods, which approximate a black-box model by an interpretable model, can help interpret the general behavior of the model. For example, Ribeiro *et al.* [9] propose a linear proxy method, *Local Interpretable Model-Agnostic Explanations* (LIME), to approximate any black-box model. Interpretation methods specific to deep learning have been recently proposed, namely gradient-based methods [10]. The model prediction is explained by backpropagating the signal from the output to the input. This type of method enables the identification of the most relevant features and neurons involved in the decision making. Several gradient-based methods are proposed in the literature including *Layerwise Relevance Propagation* (LRP) [11, 12], *Integrated Gradients* [13], and *DeepLift* [14]. In [15], the authors show that among these methods, DeepLift and LRP are better aligned with human intuition since they satisfy some desirable properties. The self-explainable models are inherently interpretable models. By definition, they include the decision trees, rules systems, sparse linear models. However, these three models generally do not perform well on high-dimensional complex data. Few works on self-explainability have been proposed for deep learning. Melis and Jaakkola [16] introduce a built-in interpretable model, *Self-Explainable Neural Network*, that behaves locally as a linear model.

A general opinion is that the black-boxes are more accurate than the self-explainable models. The capacity of interpretability is often viewed as a constraint of the model that decreases its performance. There would be a trade-off between performance and interpretability. However, recently a part of the machine learning community claims that performance and interpretability are not exclusive. Rudin [17] explains why black-box models should be avoided for crucial decisions, like in medical applications, even with the use of post-hoc interpretation. For example, the proxy methods create a new model that approximates the decision process of black-box models, leading to an imperfect fidelity in explanation. In addition, different explanations can be obtained for the same prediction using different interpretation methods or the same interpretation method with different parameters [18, 19]. Rudin, therefore, promotes the development of high-accurate self-explainable models. Self-explainable deep learning model is one of the solutions.

All of these methods, post-hoc and self-explainable, consider that the interpretation of a model consists of the identification of the inputs and the part of the model, in case of deep learning the set of neurons, that support the predictions. In the context of phenotype prediction from gene expression, these methods generally do not provide an understandable explanation. The explanation must be completed with knowledge of the domain. For example, we have to explain which biological functions are represented in the model and which ones are used to compute the patient outcomes.

Few works have been published on the construction of self-explainable neural networks based on gene expression data using prior biological knowledge. Prior knowledge comes from the ontologies such as Gene Ontology (GO) [20], Kyoto Encyclopedia of Genes and Genomes (KEGG) [21], Reactome [22], or Search Tool for the Retrieval of Interacting Genes/Proteins (STRING) [23]. Among them, the closest literature to our work [24] incorporates GO into a neural network, called *Gene Ontology Neural Network* (GONN). They replace one hidden layer by one level of the GO subontologies and connect the input features by partial connections according to the annotations with the ontologies. In this way, some input features cannot be included if there are not connected to the ontologies. Similarly, *Gene-Pathway-Disease* (GPD) [25], *Pathway-Associated Sparse Deep Neural Network* (PASNet) [26], and *Gene Regulatory network-based Regularized Artificial Neural Network* (GRRANN) [27] integrate respectively biological pathways and regulators from protein-protein/protein-gene interactions in the first layer. These architectures contain at most two hidden layers. For example, PASNet tries to capture nonlinear interactions between pathways in a second hidden layer. However, deep neural networks allow deeper representations of hierarchical relations between gene expression and biological objects. By using prior knowledge, these works first attempt to boost the model performances on their target tasks. Yet, they do not clearly show whether the neurons correspond to the associated biological concept or not. Integrating knowledge may not be so beneficial for learning.

In this paper, we propose a self-explainable deep fully-connected neural network, called *Deep GONet*, based on gene expression data. This model is constrained by prior biological knowledge from GO, which is widely used in the bioinformatics community. The architecture represents different levels of the ontology preserving the hierarchical relationships between the GO terms by using sparse regularization. Our objective is to build an accurate and relevant interpretable model for cancer detection. Each neuron is associated with a GO function and the links between these functions are represented by the network connections. A prediction of the network can, therefore, be directly explained by the set of biological functions.

The paper is organized as follows. We first describe the proposed novel model, Deep GONet, for biological interpretation. Then, the model is evaluated on two public datasets and compared with other approaches. We also provide how to obtain the explanations of outcomes and their biological significations at three levels (disease, subdisease, and patient). The conclusions to this paper and some future research directions are finally presented.

## Methods

We propose a new neural network model, *Deep GONet*, that is self-explainable and embeds the biological knowledge contained in GO. Our model is based on a MLP constrained by the GO structure. The constraints are introduced into the network using an adaptive regularization term.

### The architecture of Deep GONet

Our model takes in the input layer the gene expression profile of a patient and returns in the output layer the prediction of a phenotype of this patient. The architecture of the hidden layers represents the structure of GO. GO gathers three ontologies that respectively describe the following categories: biological process (GO-BP), molecular function (GO-MF), and cellular component (GO-CC). We chose to base the architecture of the hidden layers on the GO-BP since it provides larger processes implied by the activity of the genes, which can be more useful for phenotype prediction. However, it is possible to implement the GO-MF or GO-CC in the network architecture with the same method.

**Fig. 1:**
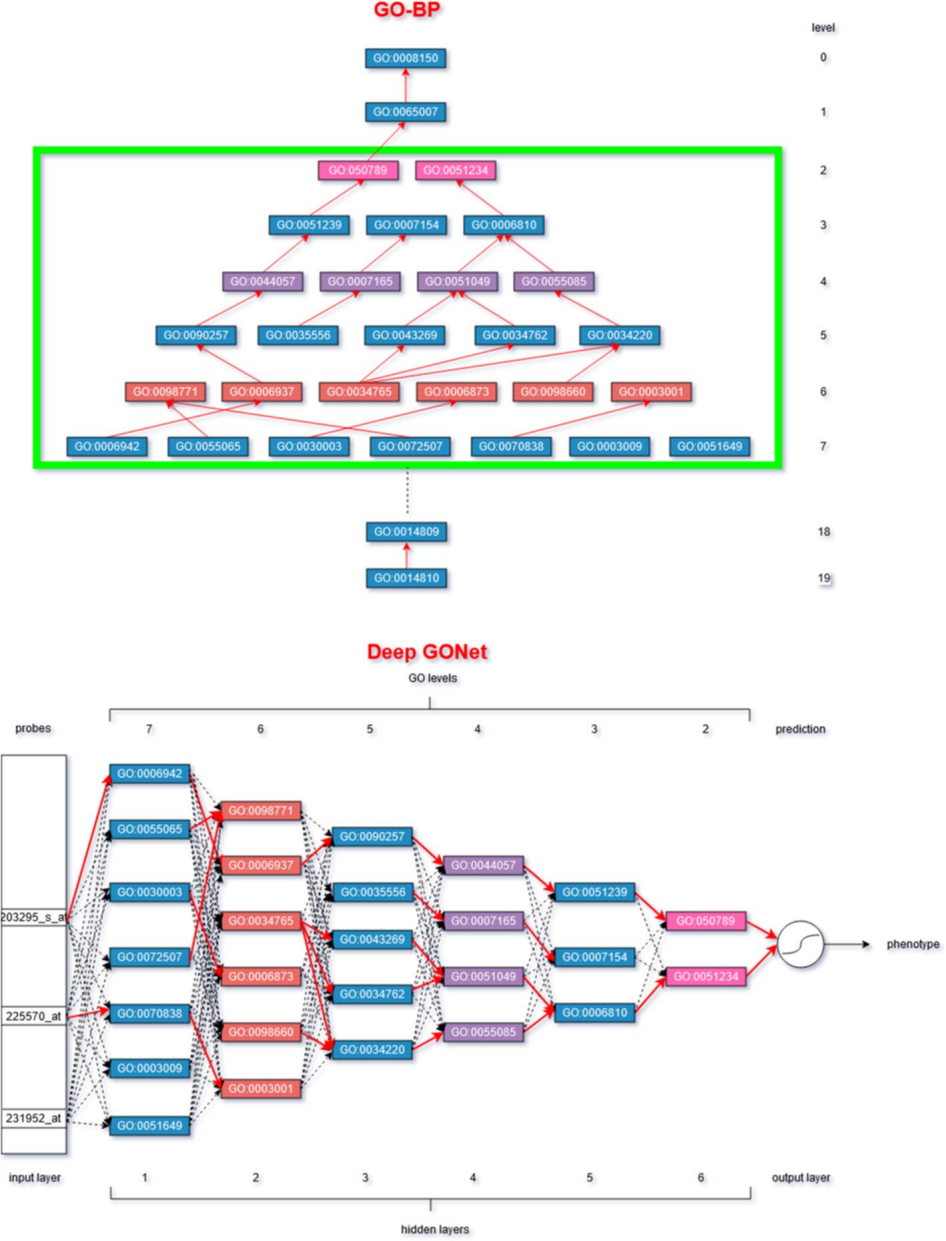
A subset of GO-BP (top) and the corresponding Deep GONet architecture (down). The green box represents the GO levels implemented in Deep GONet. The red and black dashed arrows represent respectively the GO and noGO connections

GO-BP is structured as a directed acyclic graph (DAG) containing 11991 nodes (version of October 2019) annotated with the input layer and distributed over 19 levels as illustrated in the top of Fig. 1. Each node is a GO term representing a biological function. Two GO terms are linked if their biological functions are related and the majority of these relations are “is a” relations. The GO terms are connected respecting a hierarchical bottom-up orientation. A GO term is assigned to a dedicated level according to its longest path to the root (i.e., GO:0008150). The GO terms in lower levels correspond to more specific functions, like *positive regulation of skeletal* (GO:0014810 at the 19th level), whereas the nodes in upper levels are more general functions such as the root function GO:0008150. The GO terms are also linked to genes via GO annotations. A parent GO term (i.e., destination of incoming connections) inherits, therefore, the set of genes from its children (i.e., origins of incoming connections).

Our neural network architecture represents the GO-BP, i.e., each hidden layer *l* represents a GO level *h*, each neuron a GO term, and each input variable a gene. Since the lowest levels of GO contain few very specific terms and the highest levels are very general, it seems not useful to implement the whole GO in our architecture. The selection of the levels is, therefore, part of the hyperparameters of the model to determine. In our experiments, the level 7 to level 2 have been selected as illustrated by the green box in the Fig. 1.

Our model is based on a fully-connected MLP that consists of an input layer, *L* hidden layers, and an output layer for phenotype prediction. The input layer is composed of genes or gene products (e.g., probes). A probe is a short DNA sequence targeting a region of one or several genes. It is the measure used in microarray data. Each neuron is connected to all neurons of the previous layer and all neurons of the next layer. Each hidden layer corresponds to a level in GO-BP and its neurons match all the GO terms of the target level. Note that the incorporation of the knowledge must respect the goal of the neural network to construct an abstract representation of the data through its hierarchical architecture. The first hidden layer of a neural network extracts the low-level features from the input layer, it corresponds to the lowest selected level of GO containing more specific biological functions. In the last hidden layers, the high-level features represent the most general biological functions of the highest GO levels. The bottom of the Fig. 1 illustrates where the levels 7 to 2 of GO are implemented in the architecture of the neural network. The activation of the *i*-th neuron of the hidden layer *l* can be expressed as: $a_i^{(l)} = f\left( \sum _{j=1}^{N_{l-1}} a_j^{(l-1)} w_{ji}^{(l)} + b_i^{(l)} \right) = f \left( z^{(l)}_i \right)$, where $w_{ji}^{(l)}$ is the weight of the connection from the *j*-th neuron of the layer $l-1$ to the *i*-th neuron of the layer *l*, $b_i^{(l)}$ is the bias of the *i*-th neuron of the layer *l*, $N_{l-1}$ the number of neurons in the layer $l-1$ , $z^{(l)}_i$ is the sum of signals received from the previous layer $l-1$, and *f* is the rectified linear unit function (ReLU) defined as $f(x)=\max (0,x)$. The output layer finally estimates the probability to belong to each class. For binary problem, the output layer contains only one neuron with a sigmoid function, given by $a^{(L)}=\frac{1}{1-\exp (z^{(L)})}$, returning the probability to predict the positive class. Note that for multiclass problem, the output layer should contain one neuron for each class *k* with a softmax function, defined as $a^{(L)}_k=\frac{\exp (z^{(L)}_k)}{\sum _{j=1}^{K}\exp (z^{(L)}_j)}$ where *K* represents the number of classes, to get a probability of belonging to each class.

In our fully-connected architecture, we identify two types of connections :

- connections corresponding to links in GO-BP (colored in red in Fig. 1), called GO connections;
- connections between two nodes that are not linked in GO-BP (marked by dashed arrows in Fig. 1), called noGO connections.

A probe in the input layer in Fig. 1 is connected to the neurons of the first hidden layer via a GO connection if it is associated with the corresponding GO term in the lowest chosen level of GO-BP (i.e., level 7 in Fig. 1), or via a noGO connection otherwise. Note that the neurons of the next hidden layers (i.e., 2 to 6 in Fig. 1) are not directly connected to the probes. These neurons can be indirectly connected to their probes by the propagation of gene expression through the GO connections of the previous layers. If we want to represent exactly the GO-BP, we can cut all noGO connections and keep only the GO connections in our architecture. However, GO only represents the current knowledge we have on biology. The ontologies change continuously with the outcoming of new scientific discoveries. Some links can be missing or wrong, and many genes are not associated with their right corresponding GO term. 33% of the probes from the microarray HG-U133Plus2 used in our experiments have no GO annotations (such as the probe 231952_at in Fig. 1). This means that these probes would not be connected to the neural network if we use only the GO connections. They would not be used to compute the prediction even if they bring relevant information. This situation could impact negatively the accuracy of the neural network. To deal with the errors and the incompleteness of the knowledge represented in GO, we keep all connections in our architecture (both GO and noGO connections). However, the noGO connections are penalized to favor the use of GO connections to compute the predictions.

### Learning and regularization of the network

The model is constrained by a customized regularization term, named $L_{GO}$, to favor the GO connections and penalize the noGO connections. This regularization term is defined as follows:

1 $$\begin{aligned} L_{GO}=\sum _{l=1}^L\Vert W^{(l)}\otimes (1-C^{(l)})\Vert _2^2 \end{aligned}$$

where *L* is the number of hidden layers of the neural network, $W^{(l)}$ is the weight matrix of the layer *l*, and $\otimes$ is the pointwise product. $C^{(l)}$ is the adjacency matrix that encodes the connections between the GO terms of the layer $l-1$ and *l* (i.e., the corresponding levels $h+1$ and *h* in GO-BP). More precisely, if a GO term *i* at the corresponding level *h* in GO-BP is a parent of GO term *j* from the level $h+1$, then $c_{j,i}^{(l)}=1$ else $c_{j,i}^{(l)}=0$. For the output layer, $C^{(L)}$ is a matrix of ones. The loss of our model is the sum of the common cross-entropy loss and our regularization term:

2 $$\begin{aligned} L=\sum _{i=1}^N \sum _{k=1}^K \left( -y_{i,k}\log {\hat{y}}_{i,k} \right) + \alpha L_{GO} \end{aligned}$$

where *N* and *K* are respectively the number of samples in the training set and the number of classes. ${y}_{i,k}$ is the indicator of the true class, i.e., ${y}_{i,k}=1$ when the *i*-th sample belongs to the class *k*, or 0 otherwise. Note that each sample only belongs to one class. ${\hat{y}}_{i,k}$ is the probability that the *i*-th sample belongs to the class *k* computed by the neural network. During the inference phase, we select the class with the highest probability to get the final prediction. Finally, $\alpha$ is a hyperparameter that weights the regularization term. With $\alpha$ close to 0, the regularization term vanishes, our model becomes a classical MLP without interpretation capacity. With a high value of $\alpha$, the learning algorithm focuses on the regularization term and ignores the cross-entropy. The resulting neural network represents perfectly the GO connections by cutting the noGO connections, but it has a weak prediction capacity. $\alpha$ is a crucial hyperparameter that controls the trade-off between the minimization of the cross-entropy and the loss $L_{GO}$.

## Results

### Dataset

We validate our model on two datasets. The first one comes from a cross-experimental study compiling microarray data of over 40.000 publicity available Affymetrix HG-U133Plus2 chip arrays [28]. These arrays were produced under different experimental protocols and concerned seventeen types of tissue. The dataset contains 54675 probes for 22309 samples whose 14749 (66.11%) are cancer and 7560 (33.89%) are non-cancer. We standardize it to a mean of zero and a standard deviation of one, and split it into a training set of 17847 examples (11799 cancer, 6048 non-cancer) and a test set of 4462 examples (2950 cancer, 1512 non-cancer). Note that the original proportions of cancer and non-cancer samples are preserved in each set. The training set is used to train predictive models and the test set to evaluate their performances.

**Table 1:** Number of samples by cancer types in the TCGA dataset

Cancer type	BRCA	HNSC	KIRC	LGG	LIHC	LUAD	LUSC	OV	PRAD	THCA	UCEC
#patients	1102	500	538	511	371	533	502	374	498	502	551
Frequency (%)	17.05	7.74	8.32	7.91	5.74	8.25	7.77	5.79	7.71	7.77	8.53

The second dataset is RNA-Seq data from The Cancer Genome Atlas (TCGA) combining 5982 samples of 11 cancer types and 482 normal samples from different tissues. Table 1 lists the number of cancer samples by cancer type. The number of input features is 56602. Before standardization, the data have been pre-normalized with FPKM (fragments per kilobase per million mapped reads) and transformed using $\log _2$. 80% of the dataset goes into a training set and the remaining 20% into the test set.

### Performances and sensitivity analysis

In this first experiment, we compare the performances of Deep GONet on cancer prediction from gene expression profile with the state-of-the-art. Binary classification is evaluated on the microarray dataset with a sigmoid function in the output layer whereas multiclass classification is performed on the RNA-Seq data with a softmax.

Deep GONet model is learned from the training set using a standard learning procedure. The number of layers and nodes are determined by the levels chosen in GO-BP. Different levels of GO-BP have been tested. For both datasets, we fix the architecture with levels 7 to 2 of GO-BP since it gives us the best performance. We test different values of the training hyperparameters and select the following settings. The weights and biases are initialized with He initializer. On the microarray dataset, dropout layers with a ratio of 0.6 are added after each hidden layer to reduce overfitting. The network parameters are optimized using adam with an adaptive learning rate of 0.001. On the RNA-Seq dataset, we choose the stochastic gradient descent with a momentum equal to 0.9. The number of epochs of the training is set up to 600. Different values of the hyperparameter $\alpha$, controlling the regularization term $L_{GO}$, are tested in the interval $[0, 10^{1}]$. The accuracy of the model is estimated from the test set according to the value of $\alpha$ to investigate the impact of this hyperparameter on the performance of the model. To reduce the variability of the results coming from the random initialization of the model parameters, 10 models for each value of $\alpha$ are learned with different random seeds for the initialization of the parameters.

Our method is compared with classical fully-connected networks using $L_2$ or $L_1$ regularization terms. These regularization terms apply a penalty on all the connections regardless of the type (GO or noGO). $L_2$ is the squared magnitude of the weights $L_{2}=\sum _{l=1}^L\Vert W^{(l)}\Vert _2^2$, and $L_1$ is the absolute value of the magnitude of the weights $L_{1}=\sum _{l=1}^L|W^{(l)}|$. These regularization terms are also controlled by a hyperparameter $\alpha$. In addition, a model without any regularization is tested for comparison at $\alpha =0$. Note that all these models use the same baseline described in Fig. 1. They are trained and tested with the same procedure used for Deep GONet. The accuracy of each model is estimated from the test set. All the experiments have been executed on a GPU RTX 2080Ti using Tensorflow 1.12.

**Fig. 2:**
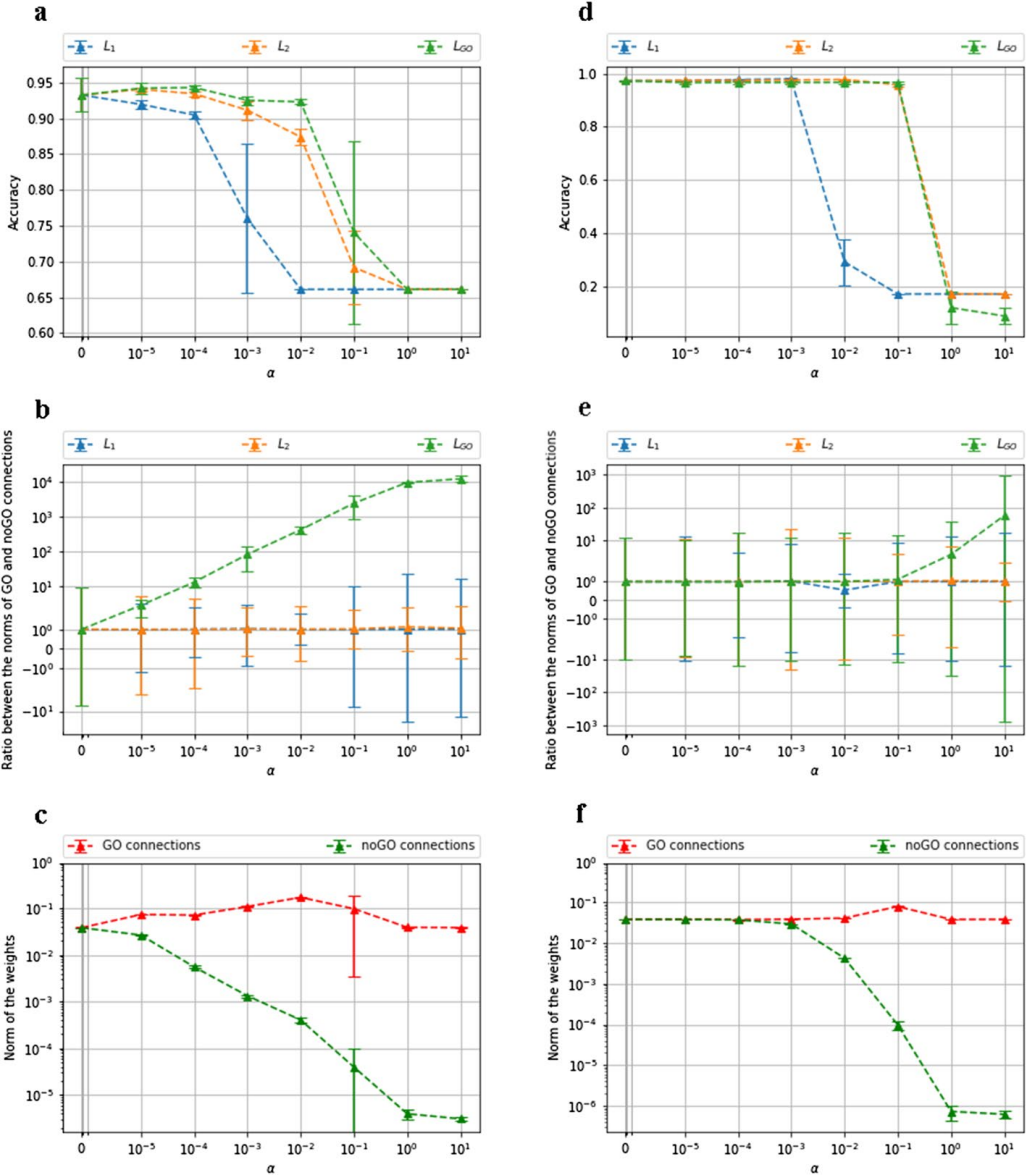
Results on the microarray dataset (left column) and the TCGA dataset (right column). **a**–**d** Accuracy of the models according to $\alpha$. **b**–**e** Ratio between GO and noGO connections weights according to $\alpha$. **c**–**f** Absolute-value norms of the GO and noGO connections from $L_{GO}$ models according to $\alpha$

In what follows, the results on the microarray dataset (Fig. 2a–c) are commented, but similar results are observed on the TCGA dataset (Fig. 2d–f).

**Table 2:** Comparison of the performances of the models on the microarray dataset

Model	Accuracy	Precision	Recall	F1-Score	MCC	AUC
RF	0.904	0.932	0.921	0.927	0.786	0.895
SVM	0.948	0.964	0.957	0.961	0.885	0.944
XGBoost	0.936	0.954	0.948	0.951	0.857	0.930
MLP	0.951	0.974	0.952	0.963	0.893	0.986
Deep GONet	0.925	0.943	0.943	0.943	0.832	0.916

**Table 3:** Comparison of the performances of the models on the RNA-Seq dataset

Model	Accuracy	Precision	Recall	F1-Score	MCC	AUC
RF	0.968	0.967	0.965	0.966	0.964	0.999
SVM	0.977	0.977	0.975	0.976	0.974	1.000
XGBoost	0.974	0.972	0.972	0.972	0.971	1.000
MLP	0.962	0.961	0.960	0.960	0.958	0.998
Deep GONet	0.970	0.970	0.967	0.968	0.967	0.998

Figure 2a (resp. Fig. 2d) plots the average and the standard deviation of the accuracy of a model with a $L_1$, $L_2$, and $L_{GO}$ penalty according to $\alpha$. The three curves begin at the same point since $\alpha =0$ corresponds to a model without regularization. We can see that the model without regularization and the one with $L_{GO}$ and $L_2$ at $\alpha =10^{-5}$ achieve the best accuracy (0.945). Note that $L_{GO}$ and $L_2$ outperform $L_1$. We also test classical machine learning methods with scikit-learn python package: Random Forest (Gini criterion, number of trees=100), SVM (linear kernel, C=1.0), XGboost (number of trees=100, learning rate=0.1), and MLP (three layers with respectively 1000, 500 and 200 nodes). Metrics performance for each method are detailed in Table 2 (resp. Table 3 for TCGA). Similar performances are obtained. These results show that our method obtains the same accuracy with the state-of-the-art algorithms, which are not self-explainable. For all models, the average accuracy decreases for high value of $\alpha$. The accuracy drops to 0.66 which corresponds to the proportion of the majority class, meaning that the models learn nothing and associate all examples to the cancer class. In this case, the regularization term takes too much importance relative to the cross-entropy. We note some special points, at $\alpha =10^{-1}$ (for $L_{GO}$ and $L_2$) and at $\alpha =10^{-3}$ (for $L_1$), with high variability. At these values of $\alpha$, some models fail to learn with an accuracy of 0.66, whereas others succeed by reaching an accuracy around 0.9. That’s why the average is between these two extremes.

In the following, we analyze the behavior of GO and noGO connections in Deep GONet and standard MLP. Figure 2b (resp. Fig. 2e) displays the ratio between the absolute-value norms of the GO (Eq. 3) and noGO (Eq. 4) connections, defined respectively as:

3 $$\begin{aligned}&\frac{1}{L}\frac{1}{\#GO}\sum _{i=1}^{L}|W^{(l)}\otimes (C^{(l)})|, \end{aligned}$$

4 $$\begin{aligned}&\frac{1}{L}\frac{1}{\#noGO}\sum _{i=1}^{L}|W^{(l)}\otimes (1-C^{(l)})|. \end{aligned}$$

For $L_{2}$ and $L_{1}$, the ratio is stuck to 1 whatever the value of $\alpha$. As expected, no distinction is made between the two types of connections. On the opposite, the ratio of the model with $L_{GO}$ regularization increases along with the growth of $\alpha$ and finally reaches its highest value of $10^4$. For this model, Fig. 2c (resp. Fig. 2f) shows the average of the absolute-value norms of the GO (Eq. 3) and noGO (Eq. 4) connections. Note that the green line in Fig. 2b (resp. Fig. 2e) is obtained from the division of the red line by the green line from Fig. 2c (resp. Fig. 2f). We can observe that the average norm of the GO connections remains between $10^{-2}$ and $10^{-1}$. In contrast, the average norm of noGO connections decreases with $\alpha$, following the accuracy trend. With $\alpha =0$ and $\alpha =10^{-5}$, the average norm of the noGO connections is very close to the one of the GO connections. The ratio between the two norms, illustrated in Fig. 2b, is below $10^1$. From $10^{-4}$ to $10^{1}$, the gap between the two norms becomes larger. The norm of noGO connections is converging almost to 0, leading to a ratio of $10^1$ at $\alpha =10^{-4}$ and the highest ratio of $10^4$ at $\alpha =10^1$. As a consequence, $L_{GO}$ seems to penalize well the noGO connections with high value of $\alpha$. At $\alpha =10^1$, the model is equivalent to a model containing only GO connections, all noGO connections are set to 0, respecting the hierarchy of GO scrupulously. However, the accuracy curves in Fig. 2a show that with a large value of $\alpha$, the model is not able to learn anymore. It means that some noGO connections are necessary for accurate predictions. In particular, the flexibility brought by the fully-connected architecture makes it possible. This advantage will be further inspected in the next sections.

In summary, imposing a number of layers and neurons is not enough to make the model interpretable. An appropriate regularization term should be added to the loss function to constrain it along with biological knowledge. If the regularization term is not customized, the GO and noGO connections will be considered identically like with a $L_2$ or $L_1$ regularization. This results in a non-interpretable model without any prior knowledge. Our model Deep GONet reaches similar prediction performances than the state-of-the-art, in both (i) penalizing properly the noGO connections, and (ii) privileging enough the GO connections to let the major information flow by them.

On the microarray dataset, the models at $\alpha =10^{-2}$ achieve an average accuracy around 0.92 and an average ratio of $10^3$. Since they represent a good trade-off between noGO connections penalization and accuracy, we analyze in-depth and interpret biologically one of the models learned with $\alpha =10^{-2}$ in the rest of this paper. The study will focus on the microarray dataset, but similar analyses can be conducted on the other dataset.

### Analysis of the Deep GONet architecture

**Table 4:** Details about the architecture of Deep GONet

Layer	Input	1	2	3	4	5	6	Output	Total
Level GO-BP	–	7	6	5	4	3	2	–	6
#neurons	54675	1574	1386	951	515	255	90	1	4772
#connections	–	86M	2.2M	1.3M	490K	131K	23K	90	90.1M
#GO connections	–	43504	1709	1585	1010	491	175	–	48K

The first part of this analysis is to check that the architecture of the model chosen in the previous section is very close to the subhierarchy of GO-BP. This model has been learned with $\alpha =10^{-2}$ and reaches an accuracy of 0.925 (reported in Table 2) as well as a ratio between GO and noGO connections around $10^{3}$. Table 4 presents in detail its architecture. The first two rows summarize the corresponding levels from GO-BP and the number of neurons from the input layer to the output one (see Fig. 1). The last two rows give for each layer the number of incoming connections (GO and noGO) and the number of incoming GO connections. Note that the total number of connections plus the number of neurons constitute the number of parameters of the model (i.e., around 90.105M). The number of connections decreases through the layers because the number of neurons by layer becomes smaller. This table shows that the large majority of the connections are noGO connections, only 0.05% are GO connections (i.e., around 48K).

**Fig. 3:**
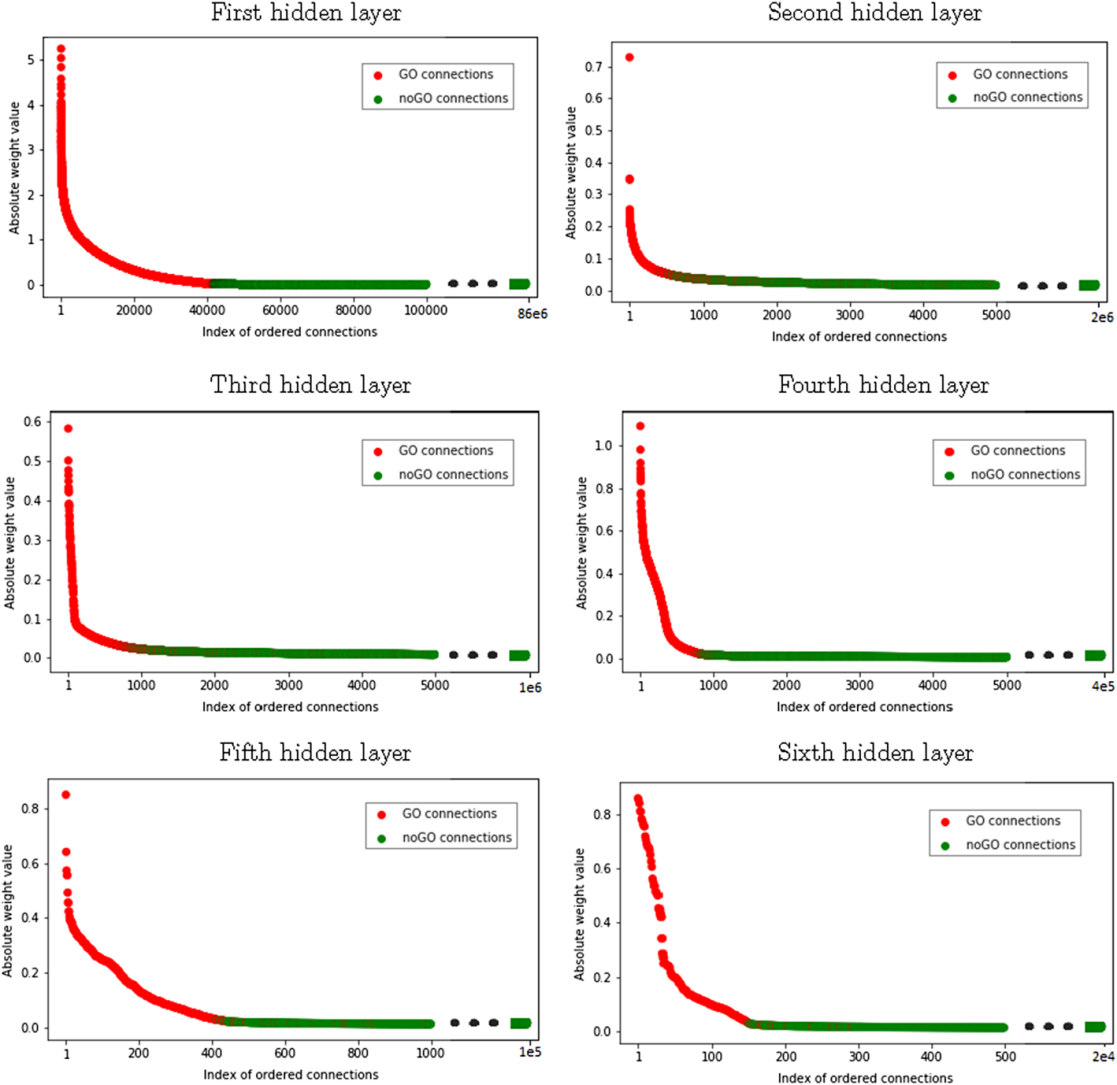
Sorting of incoming connections from each layer according to their absolute weight value

Figure 3 displays for each layer the sorting of the incoming connections according to the absolute value of their weight. The incoming GO (resp. noGO) connections are colored in red (resp. green). We first note that the connection matrices are very sparse, few connections have their weight significantly different from 0. This means that the gene expression is not uniformly propagated through the entire network and only a small part of the network is useful for the prediction. For all hidden layers, most of the GO connections are ranked before the noGO connections. Some of the GO connections can have a very high weight (around $10^0$). The high-weighted incoming GO connections of a neuron promote the activation of its corresponding function. The value of the noGO connections is close to 0 as expected by the application of the $L_{GO}$ penalization. Some GO connections are ordered at the bottom of the rank. For example, the 43 505th GO connection of the first layer is ranked 33 041 190th. The GO connections, which do not seem to be useful for the network, get a very low value ($7.10^{-6}$ for our example). On the opposite, despite the application of the $L_{GO}$ penalty on noGO connections, few of them have higher weight than GO connections as illustrated in the figure of the second hidden layer. These results show that the architecture of our model is very close to the GO-BP architecture since most of the weights of noGO connections are set to 0. The rare noGO connections with high weight are interesting. It represents links that the network has to build to compute accurate predictions. It would be interesting to investigate which GO terms or probes that have been connected by these noGO connections.

The next analyses of our network will be based on two sets of values: the neurons activation and neurons relevance. The activation $a_i^{(l)}$ of a neuron *i* from layer *l* gives information about how much signal $z_i^{(l)}$ flows from this neuron. However, high activation doesn’t necessarily mean that the neuron contributes highly to the prediction. A neuron highly activated by a given sample may have outcoming connections with very low weight. In this case, it will contribute a few to the prediction. Therefore, to identify which neurons and connections are used to compute the predictions, we employ a gradient-based method, *Layerwise Relevance Propagation* (LRP) [11, 12]. The aim of LRP is to retropropagate the output signal of one sample from the upper hidden layer to the input layer. A relevance score assigned to a neuron *i* of a layer *l* is given by

5 $$\begin{aligned} R^{(l)}_i=\sum _{j=0}^{N_{l+1}}\frac{a_i^{(l)}w_{i,j}}{\sum _{k}a_k^{(l)}w_{k,j}+\epsilon }R^{(l+1)}_{j} \end{aligned}$$

where $\epsilon$ is a factor of stabilization (equals to $10^{-7}$ in our experiments), $R^{(l+1)}_{j}$ is the relevance of a neuron *j* of the upper layer $l+1$, and $R^{(L)}=z^{(L)}$. This score represents the proportion of the output signal passing through the neuron and its outcoming connections. The relevance of a neuron represents its importance in the computation of the prediction. For each patient, we can get a relevance (resp. activation) profile by layer composed of neurons relevance (resp. activation). An analysis of the neurons relevance of each layer confirms the fact that only a small subset of neurons is important to compute a given prediction.

### Biological significance of the neurons

**Fig. 4:**
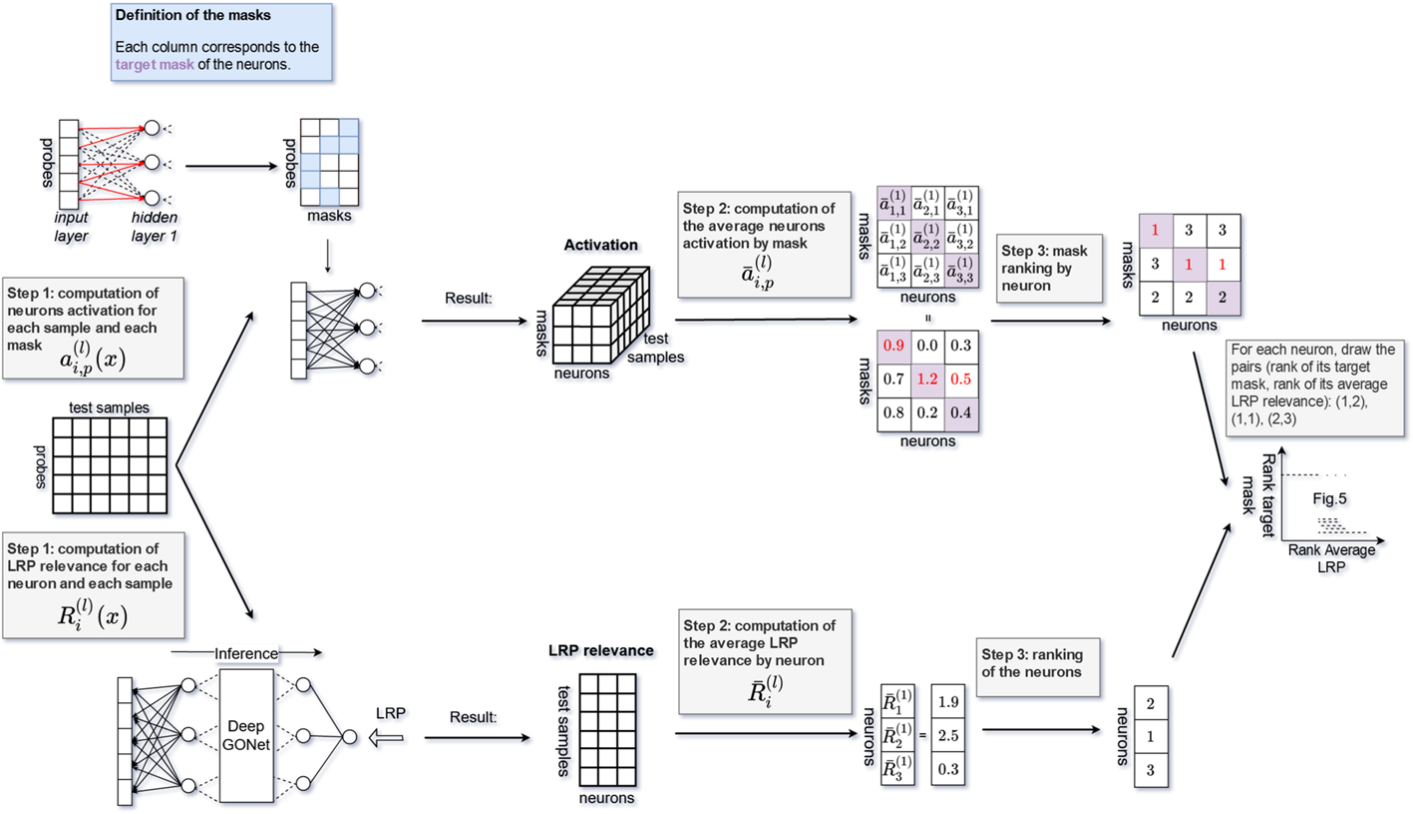
Illustration of the procedure to evaluate the biological significance of neurons from the first layer. The upper part shows how to calculate the rank of the target mask of each neuron. The lower part shows how to compute the rank of the neurons according to their LRP relevance. The relationship between these two metrics is evaluated through a final figure (e.g., Fig. 5)

In this section, we check that the neurons of our network actually represent their corresponding GO term, i.e., the activation of a given neuron represents the expression of the corresponding biological function. For that, we use the fact that each GO term in GO is associated with a set of probes. If a given neuron really represents its corresponding GO term, the set of probes associated with this GO term should activate the neuron more than any other set of probes. We propose a procedure illustrated in Fig. 4 based on this principle to test the biological significance of the neurons and evaluate the relationship with the importance of the neurons by using LRP with the package innvestigate [29]. We detail in the following only the analysis of the first hidden layer. However, we can apply similar analyses to the other layers.

**Fig. 5:**
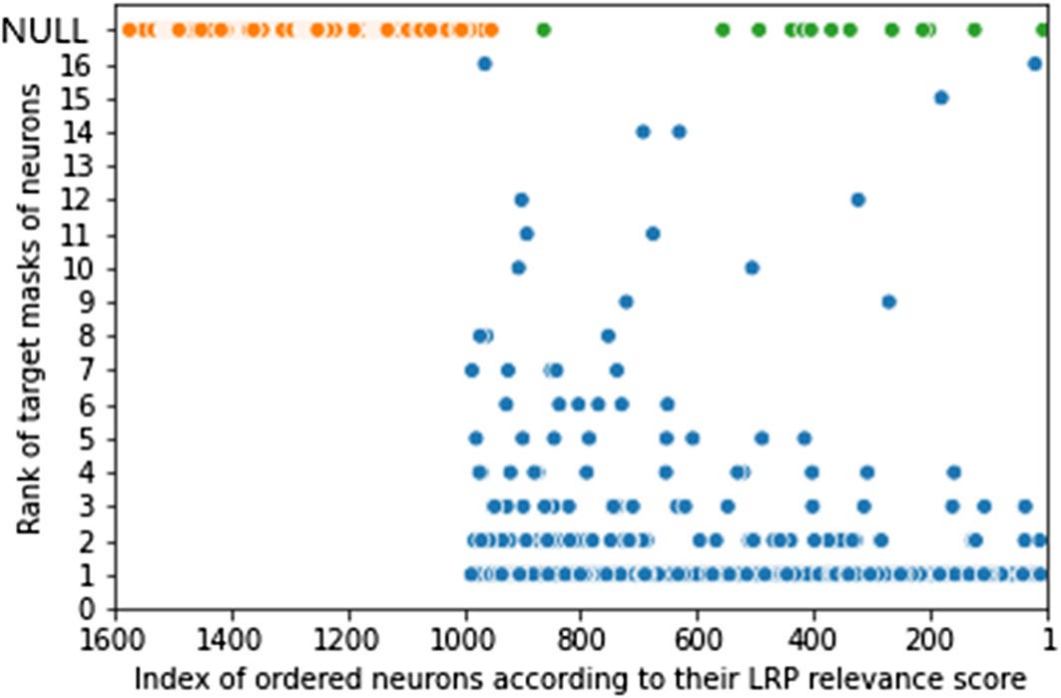
Sorting of neurons from the first hidden layer according to the rank of their target mask (*y*-axis) and their LRP rank (*x*-axis)

The first hidden layer contains 1574 neurons connected to the input layer. Each GO term is connected to a set of probes (median: 8, max: 1357, min: 1). Regarding this information, the target mask of a neuron is defined as follows:

- all the probes of the input layer, which are not connected to the GO term, are set to be 0;
- the values of the remaining probes in the set are unchanged.

In total, we have 1574 masks because none of the neurons has the same target mask. For every neuron, all these masks are applied to the input layer to identify whether the neuron is activated more by its target mask than the other masks. This can be measured by the rank of the target mask. The following procedure, illustrated in the top of Fig. 4, details how to get the rank of the target mask for each neuron in a layer *l*:

- *Step 1*: For each sample *x* from the full test set, the activation $a^{(l)}_{i,p}(x)$ of each neuron *i* for a given mask $m^{(l)}_p$ is calculated where $p=1,\ldots ,i,\ldots ,N_l$. As a neuron and its target mask share the same index, the activation of a neuron *i* for its target mask is $a^{(l)}_{i,i}$. Note that there is no bias due to the length of the mask.
- *Step 2*: Then, the average value of these activations ${\bar{a}}^{(l)}_{i,p}$ is considered. For example, assuming that there are 3 neurons (3 masks) in the first hidden layer, for neuron 1, we obtain ${\bar{a}}^{(1)}_{1,1}=0.9$, ${\bar{a}}^{(1)}_{1,2}=0.7$, and ${\bar{a}}^{(1)}_{1,3}=0.8$.
- *Step 3*: For each neuron, its activation values of all the masks are ordered in a decreasing way. Then, we have the rank ${\bar{a}}^{(1)}_{1,1}, {\bar{a}}^{(1)}_{1,3}, {\bar{a}}^{(1)}_{1,2}$. It means that the neuron 1 embodies the corresponding GO term because the rank of its target mask is 1.

We compare the rank of the target mask of the neurons with the rank of the neurons according to their LRP relevance. The computation of this rank, described in the bottom of the Fig. 4, follows the steps 1 to 3 without considering the masks. For each sample *x* and all the neurons *i* in a layer *l*, $R^{(l)}_i(x)$ is computed, then the average across the samples ${\bar{R}}^{(l)}_i$ is calculated. For example, we acquire ${\bar{R}}^{(1)}_1=1.9$, ${\bar{R}}^{(1)}_2=2.5$, and ${\bar{R}}^{(1)}_3=0.3$ respectively for the neurons 1, 2, and 3. According to Step 3, the relevance scores are ordered in the following sequence ${\bar{R}}^{(1)}_2, {\bar{R}}^{(1)}_1, {\bar{R}}^{(1)}_3$. Then, based on this sequence, a rank to each neuron is attributed: neuron 2 gets the rank 1, and so on. Figure 5 plots the rank of the target masks of the neurons along y-axis and the rank of the neurons according to their LRP relevance along x-axis. Note that the value of the ranks is up to the total number of neurons (i.e., 1574).

For the y-axis, a rank can get a NULL value or a discrete value in the range [1,16]. On the one hand, a NULL rank means that the activation of a neuron for its target mask is zero, which concerns 603 neurons (i.e., 38.31% of the 1574 neurons). Specifically, in total 591 neurons have a zero activation value for any mask and generally a LRP rank above the 1000-th order (colored in orange in Fig. 5). The rest 12 neurons are activated by at least one another mask, and their LRP rank is below 1000-th order (colored in green in Fig. 5). On the other hand, there exist 971 neurons (61.69%) that have a positive activation for their target mask and show higher ranks, below order 1000. Among the 971 neurons, the target masks of 850 neurons rank 1, the other 121 neurons rank between 2 and 16. In conclusion, most of the neurons, which contribute highly to the prediction (LRP rank below order 1000), are well ranked for their target mask. This means that the important neurons for the prediction mainly match with their corresponding GO term.

Concerning the neurons with a NULL rank for their target mask, the major part has low LRP relevance. These neurons are not important for the predictions, they will not be, therefore, used in the interpretation. The associated GO terms can be ignored. However, the few neurons that have a high LRP relevance and a NULL rank are much more interesting (colored in orange in Fig. 5). For instance, the neuron associated with GO:0071644 (*negative regulation of chemokine (C-C motif) ligand 4 production*) has a LRP rank of order 15, but it is not activated by its target mask. Its target mask is composed of 2 probes, linked by GO connections weighted 0.1 and 0.04 respectively. On the opposite, 890 of the 1000 first noGO connections from the input layer, which have the same value with GO connections of norm-1 0.01, point this neuron. Since these neurons are not activated by their target mask, we cannot conclude that they are associated with their corresponding GO term. We note that a large part of the noGO connections with high weight is connected to these neurons. Moreover, these noGO connections connect mainly probes with no annotation in GO, i.e., probes that have only noGO connections. We can assume that the network distorts these neurons from their primary use to propagate the information of probes without GO annotations via noGO connections. These neurons do not represent anymore their corresponding GO terms but an unknown biological information useful for the predictions.

### Biological interpretation of the results

In this section, we show how to propose relevant biological interpretations of the model Deep GONet and its predictions. We provide three levels of interpretation: the disease level, the subtype of disease level, and the patient level. First, we study how our model detects cancer from heterogeneous samples basing on the neurons activation. Then, we analyze independently a subtype of cancer by extracting a subnetwork associated to it from the relevance scores computed by LRP. We finally present how the individual prediction of a patient can be explained.

#### Model interpretation at disease level

**Fig. 6:**
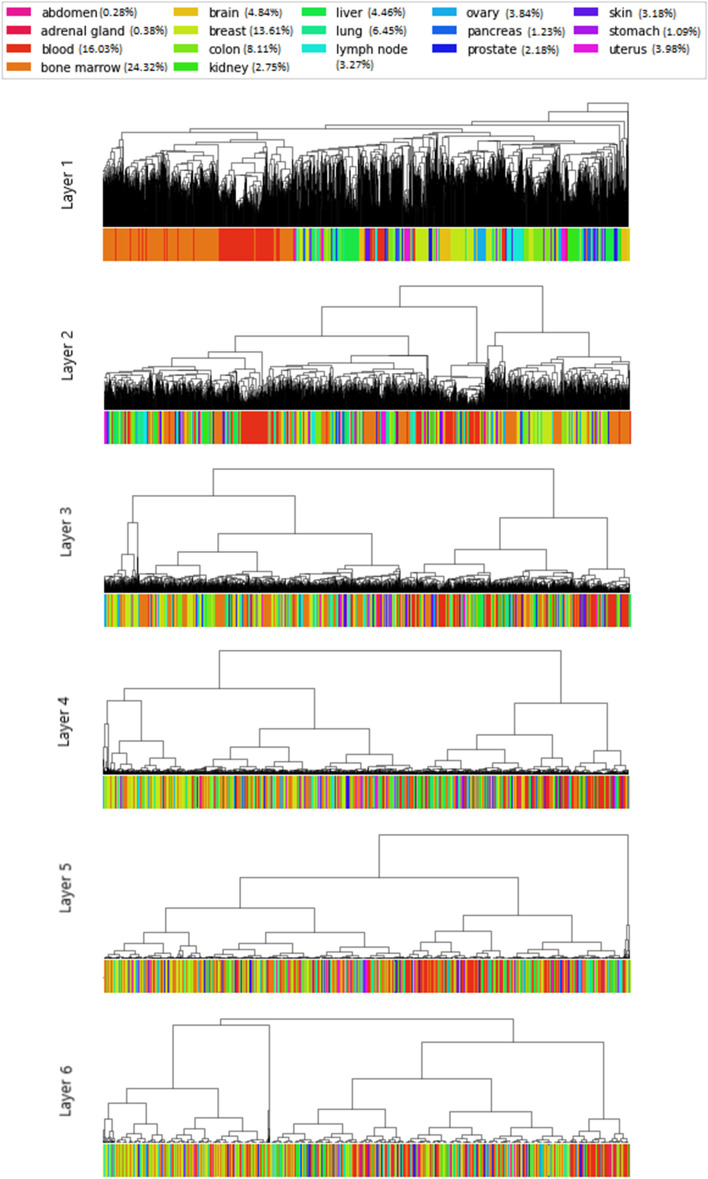
Hierarchical clustering of test samples correctly predicted as cancer based on their activation profiles. Dendrograms are displayed by layer from the first hidden layer (top) to the sixth hidden layer (bottom)

In this subsection, we study the clustering of samples correctly predicted as cancer according to their activation profiles. For each sample, an activation profile constituted of the activation of all neurons is computed during the forward pass. For each layer, we define an activation matrix of size $(N,N_{l})$ containing the activation of all neurons of this layer for all samples, where *N* is the number of samples, and $N_{l}$ the number of neurons in layer *l*. From these activation matrices, we perform hierarchical clustering using the average linkage and the euclidean distance. The dendrograms of each layer are plotted in Fig. 6. The colors on the dendrogram represent the type of tissue of the samples. In the dendrogram of the first hidden layer, we see that the patients from the same tissues tend to be grouped into the same clusters. It is especially the case for bone (colored in orange), blood (colored in red), and lymph node (colored in cyan). Tissues of the same type tend to share the same activation profiles, meaning that some neurons and their corresponding GO terms are dedicated to one tissue. This clustering according to the tissue is still present in layer two although it is less significant. From the third hidden layer, the clustering of samples from the same tissues becomes less clear. From layer four to six, the clusters contain samples from different tissues. This means that the same GO terms are activated for cancer prediction regardless of the characteristics of the inputs (such as the localization of the cancer). A signature of cancer shared by all tissues has been learned in the last layers of the network. In conclusion, according to the way our architecture is structured, the lower hidden layers gather more specific GO terms. The associated neurons are responsible to extract cancer features particular to a type of tissue. The model being more general incrementally, the upper hidden layers are instead in charge of extracting common cancer features to any type of tissue. It shows that our classifier is universal and able to extract the features shared by various cancers through common GO terms. In the last hidden layer, the existence of several clusters indicates that different neurons are activated to provide the same prediction since the signal from the input layer to the output layer propagates along different paths. Note that this capacity of extracting specific patterns in the first layers and generic patterns in the last layers is well-known in the deep learning models, which has been widely studied in the convolutional neural networks for image analysis [30].

Through the activation profiles, we see how the information is flowing in the general cancer network. In the next analyses, we will focus on neuron importance by using the relevance score. It’s a better indicator for more granular results (e.g., at subdisease and patient level) to assess exactly which neurons contribute the most to individual sample’s outcome.

#### Model interpretation at subdisease level: breast cancer

In this subsection, we show how to interpret biologically our model for a specific subtype of cancer.

**Fig. 7:**
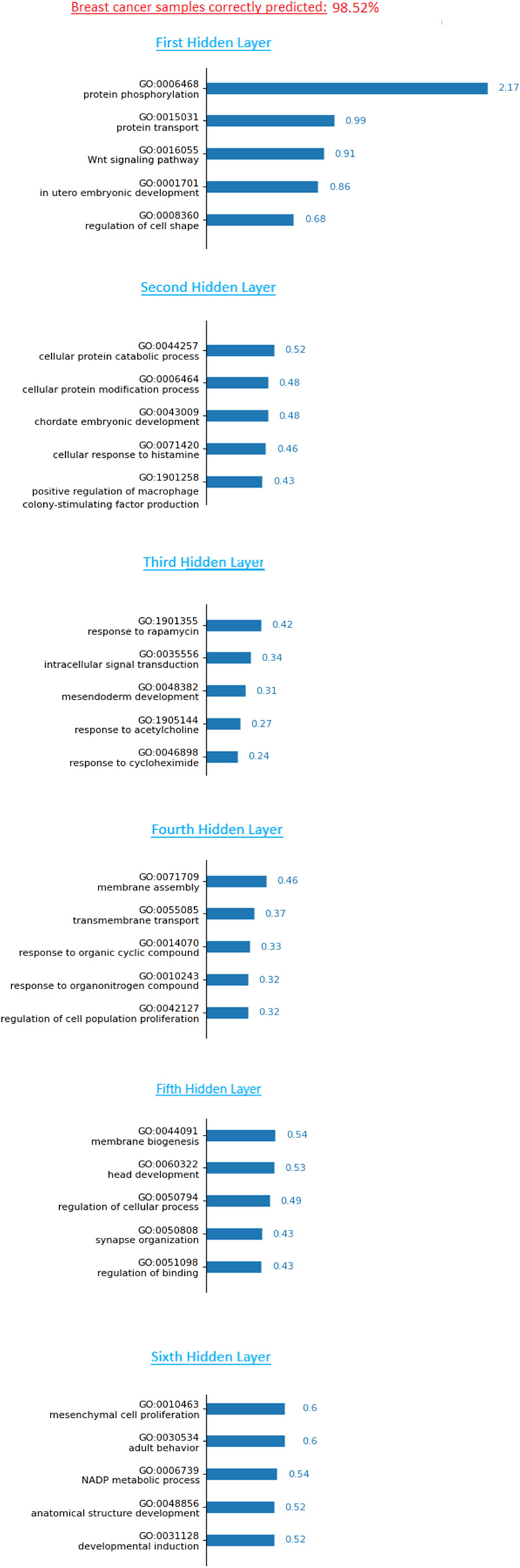
Interpretation of a subnetwork for breast cancer. The GO terms are ordered according to their relevance score for each layer. The top-5 GO terms are given with literature support

To this aim, we propose a tool that points out the main biological functions used for cancer predictions and quantify their contribution. Similarly as shown in Fig. 4, we first compute the average LRP relevance of each neuron across a type of cancer samples from one tissue of interest. Then, for each layer, the neurons are sorted according to their relevance score and the most important ones are returned with their corresponding GO term and biological function. In Fig. 7, we give an example on the breast cancer. For each hidden layer, the five most important biological functions are reported with their LRP relevance. Note that this tool can also be used to determine which probes or genes are the most involved in the predictions.

This interpretation tool can be completed by a manual search in the literature in order to identify the links between the returned functions and the predicted phenotype. Biological and medical experts can, therefore, judge the relevance of the prediction based on this final interpretation. Among all GO terms supporting the prediction of cancer in this subnetwork, some of them are known to be related to cancer. The first hidden layer shows two terms (GO:0015031, GO:0006468) linked to protein activities that can highlight a high activity of protein disorder. In the second layer, both GO:0071420 and GO:1901258 reflect the immune activity response to cancer [31, 32]. The macrophage colony-stimulating factor is among one of the growth factors overexpressed in many tumors. Two additional terms related to protein (GO:0044257, GO:0006464) are present. On the third hidden layer, we have GO:0035556 encoding the biological function *intracellular signal transduction*, part of cell communication. Intracellular signal transduction is a chain of biochemical reactions transmitting signals from the cell surface to receptors of various components within the cell. It finally ends with a cellular response as a cell state change, i.e., cell growth and many other processes. It was found that hyperactivity of these signal pathways can increase the proliferation of abnormal cells [33]. In the fourth hidden layer, GO:0042127 introduces the uncontrolled proliferation well known in tumor spread. Between the fourth and fifth hidden layers, different GO terms refer to membrane activity (GO:0071709, GO:0055085, GO:0044091). Many alterations of the membranes of tumor cells have been detected such as depolarisation [34]. GO:0050794 can point to the deregulation of cellular processes. Finally, concerning GO:0006739 in the last hidden layer, studies show that the quantity of this molecule can be much higher in cancer cells [35].

Note that if our objective had been to predict a subtype of cancer, the interpretation and the subnetworks extracted would be different. The interpretation of a model depends strongly on the phenotype prediction problem.

#### Prediction interpretation of a given patient

**Fig. 8:**
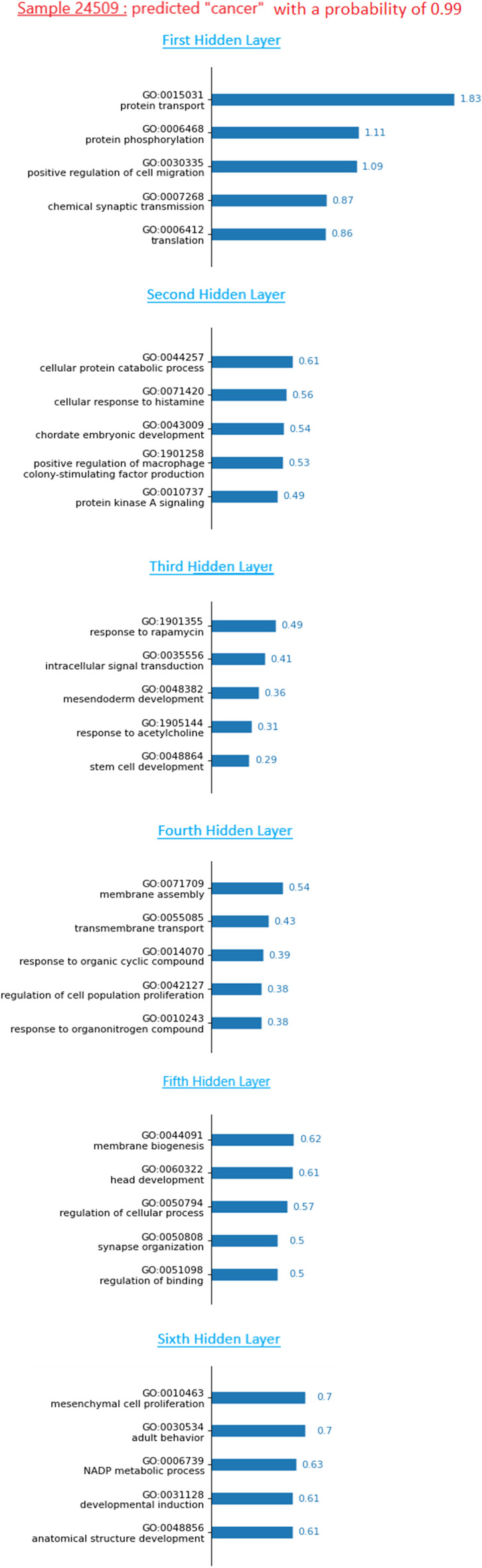
Interpretation of the prediction of the sample 24509. The GO terms are ordered according to their relevance score for each layer. The top-5 GO terms are given with literature support

In this subsection, we show how to provide a biological interpretation of the predicted outcome of one single patient. The objective is to propose a tool to the physicians and scientists that makes understandable the prediction computed by the model for a patient. After our model predicts the outcome of a patient with a probability score, the LRP relevance of each neuron is computed. We can then apply the tool previously presented to obtain a rank of neurons by layer. Figure 8 shows an example of the biological interpretation that we propose. In this example, we explain the prediction of the patient 24509 predicted cancer by Deep GONet with a probability of 0.99. Note that this patient is from the breast cancer previous subset. As in Fig. 7, the top-5 important neurons are reported with their relevance score.

In the example of Fig. 8, in the first hidden layer, the term GO:0030335 can highlight the phenomenon of cancer cell invasion into surrounding tissues, which characterizes the beginning of tumor metastasis [36]. In the second hidden layer, GO:0010737 coding for *protein kinase A signaling* can refer to some dysregulations or mutations of the protein specie which contribute to all stages of cancer development [37]. In the third layer, the top-5th term (GO:0048864) can inform the production of cancer stem cells that have similar characteristics with normal stem cells. For the next layers, we find the same relevant GO terms from the previous biological interpretation at subdisease level. We can notice that especially in the first layers (one to three), there can be some differences in the most important GO terms for the prediction between one patient and patients from the same subdisease. In this way, we can identify patients that have distinct characteristics from the average.

We also observe that less than 1% of non-cancer samples have LRP relevances higher than those of the neurons in Fig. 8. It confirms that these neurons extract patterns characteristic of cancer in relation with the biological functions (tumor cell proliferation, protein disorder...).

## Discussion

We point out that the goal of the interpretation is to explain how the model works and not how the biology works. Sometimes, there are no obvious relations between the biological functions, returned by the interpretation, and predicted phenotype. This does not necessarily mean that the predictions are not reliable. We remind that a model looks for correlations between the output and the input and not for causalities. When a function, which seems not related to the phenotype, is returned, it is possible that this function either has an indirect correlation or is linked by an unknown causality relation with the phenotype. However, the more biological functions returned by the interpretation are coherent with the phenotype, the more we can trust the model predictions. If the most part of the interpretation is incoherent with the current biological knowledge, the reliability of the model should be interrogated. The model may overfit or be mislead by a bias in the training set.

Although the model interpretation is not a tool for biological discovery, some parts of our neural network could be investigated in this way. We refer, in particular, on the high-weighted noGO connections and neurons diverted from their GO term. These elements connect to the network the probes that have not annotations. It could be interesting to understand why these probes have been used for prediction, they should be related to the phenotype. We could also investigate how the expression of these probes is combined into the hidden layer. The probes connected to the same neuron could have close biological functions related to the predicted phenotype. Our model can, therefore, help enrich GO by raising new hypotheses that have to be validated with further biological experiments.

## Conclusion

In this paper, we propose, Deep GONet, a new self-explainable deep learning model for phenotype prediction based on gene expression data. We demonstrate that its prediction performances are equivalent to classical deep learning and machine learning methods. The whole architecture of Deep GONet is interpretable and easy-understandable by biologists since it reflects the knowledge that they usually employ. Each layer of Deep GONet corresponds to one level of GO and each neuron to a GO term. The addition of a customized regularization $L_{GO}$ helps the model to better respect this knowledge by focusing on the real connections between the biological objects. The experiments presented on cancer detection show how to provide easily an interpretation of the model and its predictions, understandable by physicians and biologists. In this paper, the architecture of Deep GONet is based on GO-BP, but any other ontologies structured as a DAG (such as GO-CC, GO-MF) can be implemented in the neural network with the same approach. In addition, the model can be applied to other gene expression datasets, or other prediction tasks such as predicting the type of cancer or the prognostic, but it requires a retraining of the model.

In future works, we plan to improve Deep GONet by adding neurons to deal with the genes without GO annotations and a second branch representing the pathways to enrich the biological interpretations. We will finally investigate the links between the activation of a neuron and the activation of the corresponding biological function.

## Data Availability

The microarray dataset is accessible from the ArrayExpress database under the identifier E-MTAB-3732. The TCGA datasets can be downloaded from the Genomic Data Commons (GDC) data portal. Deep GONet is implemented in Python using the Tensorflow framework. The code is available at https://forge.ibisc.univ-evry.fr/vbourgeais/DeepGONet.git.

## Abbreviations

BRCA: Breast invasive carcinoma
DAG: directed acyclic graph
GO: Gene Ontology
GO-BP: Biological Process Ontology
GO-CC: Cellular Component Ontology
GO-MF: Molecular Function Ontology
HNSC: Head and neck squamous cell carcinoma
KIRC: Kidney renal clear cell carcinoma
LGG: Brain lower grade glioma
LIHC: Liver hepatocellular carcinoma
LRP: Layerwise Relevance Propagation
LUAD: Lung adenocarcinoma
LUSC: Lung squamous cell carcinoma
MLP: multilayer perceptrons
OV: Ovarian serous cystadenocarcinoma
PRAD: Prostate adenocarcinoma
SVM: support vector machine
TCGA: The Cancer Genome Atlas
THCA: Thyroid carcinoma
UCEC: Uterine corpus endometrial carcinoma
